# Papillary and medullary thyroid carcinoma with a single hybrid lymph node: a case report with review of literature

**DOI:** 10.1093/jscr/rjaf389

**Published:** 2025-06-16

**Authors:** Ari M Abdullah, Shaho F Ahmed, Rawa M Ali, Aras J Qaradakhy, Abdulwahid M Salih, Aso S Muhialdeen, Hardi M Dhahir, Abdullah A Qadir, Shko H Hassan, Fahmi H Kakamad

**Affiliations:** Scientific Affairs Department, Smart Health Tower, Madam Mitterrand Street, Sulaymaniyah 46001, Iraq; Department of Pathology, Sulaymaniyah Teaching Hospital, Zanko Street, Sulaymaniyah 46001, Iraq; Scientific Affairs Department, Smart Health Tower, Madam Mitterrand Street, Sulaymaniyah 46001, Iraq; Scientific Affairs Department, Smart Health Tower, Madam Mitterrand Street, Sulaymaniyah 46001, Iraq; Department of Pathology, Hospital for Treatment of Victims of Chemical Weapons, Mawlawy Street, Halabja 46018, Iraq; Scientific Affairs Department, Smart Health Tower, Madam Mitterrand Street, Sulaymaniyah 46001, Iraq; Department of Radiology, Shorsh Teaching Hospital, Shorsh Street, Sulaymaniyah 46001, Iraq; Scientific Affairs Department, Smart Health Tower, Madam Mitterrand Street, Sulaymaniyah 46001, Iraq; Department of Surgery, College of Medicine, University of Sulaimani, Madam Mitterrand Street, Sulaymaniyah 46001, Iraq; Scientific Affairs Department, Smart Health Tower, Madam Mitterrand Street, Sulaymaniyah 46001, Iraq; Scientific Affairs Department, Smart Health Tower, Madam Mitterrand Street, Sulaymaniyah 46001, Iraq; Scientific Affairs Department, Smart Health Tower, Madam Mitterrand Street, Sulaymaniyah 46001, Iraq; Scientific Affairs Department, Smart Health Tower, Madam Mitterrand Street, Sulaymaniyah 46001, Iraq; Scientific Affairs Department, Smart Health Tower, Madam Mitterrand Street, Sulaymaniyah 46001, Iraq; Department of Surgery, College of Medicine, University of Sulaimani, Madam Mitterrand Street, Sulaymaniyah 46001, Iraq; Kscien Organization for Scientific Research (Middle East Office), Hamdi Street, Azadi Mall, Sulaymaniyah 46001, Iraq

**Keywords:** papillary thyroid carcinoma, medullary thyroid carcinoma, mixed lymph node metastasis, synchronous

## Abstract

Synchronous medullary and papillary thyroid carcinoma (MTC-PTC) is rare, particularly with metastasis to the same lymph node. This study presents a unique case of synchronous metastatic MTC and PTC in a single lymph node. A 35-year-old male presented with a neck swelling, diagnosed with MTC-PTC. Imaging revealed thyroid nodules, and histopathological examination following thyroidectomy confirmed mixed MTC-PTC with lymph node metastasis. Literature reports similar cases in patients aged 24–64, often presenting with neck lumps. Management typically involves total thyroidectomy with lymph node dissection, yielding favorable outcomes. Treatment should follow guidelines based on disease stage. Due to its rarity, further research is needed to better understand and manage this condition.

## Introduction

Thyroid carcinoma, comprising <1% of all malignancies, includes papillary (PTC) and medullary thyroid carcinoma (MTC) as the most common subtypes, each exhibiting distinct molecular and clinical profiles [1–3]. Rarely, these subtypes co-occur as mixed MTC-PTC, with a reported higher incidence in females and unclear pathogenesis, potentially linked to genetic factors [4]. The coexistence of both tumor types in lymph nodes complicates the understanding of metastasis and staging. Accurate diagnosis is critical, with treatment primarily targeting the medullary component. This study presents a rare case of synchronous metastatic MTC and PTC in a single lymph node, highlighting diagnostic and therapeutic challenges. The report follows the CaReL guidelines and includes a literature review [5].

## Case presentation

### Patient information

A 35-year-old male presented with an anterior neck swelling. The patient’s past medical and surgical history was unremarkable. However, his father and two sisters had previously undergone total thyroidectomy and were diagnosed with MTC.

### Clinical findings

On examination, the patient had stable vital signs. The thyroid gland was enlarged (Grade 2–3), with associated cervical lymphadenopathy. The neck swelling had progressively increased over the past 8 months, was non-tender, and was accompanied by mild dysphagia to solids and occasional hoarseness. There were no signs of skin discoloration, ulceration, venous engorgement, respiratory distress, or catecholamine excess, and the neurological and systemic examinations were unremarkable.

### Diagnostic approach

Routine laboratory tests for thyroid functions showed the following results: thyroid-stimulating hormone at 1.13 mIU/L, free T4 at 18.3 ng/dl, calcitonin at 1970 pg/ml, parathyroid hormone at 27.5 pg/ml, serum calcium at 9.85 mg/dl, and carcinoembryonic antigen (CEA) at 69.18 ng/dl. The significantly elevated calcitonin and CEA levels strongly suggest MTC. The neck ultrasound (US) revealed a well-defined, lobulated, solid, hypoechoic nodule measuring 28 × 19 × 15 mm in the mid-upper third of the left thyroid lobe, classified as TIRAD 5. Additionally, a smaller 5 × 3 × 3-mm TIRAD 3 nodule was noted on the left lobe, and a solid, hypoechoic, TIRAD 5 nodule measuring 15 × 12 × 10 mm was found in the middle third of the right lobe, showing both micro- and macrocalcification. There were several suspicious lymph nodes in the central and left lateral cervical groups, the biggest one measuring 10 × 6 mm and located below the left lower pole, while the next two largest ones were 16 × 5 mm and 8 × 7 mm. A computed tomography (CT) scan with intravenous contrast covering the neck, chest, pelvis, and abdomen revealed multiple coarse calcifications in both thyroid lobes, along with bilaterally enlarged lymph nodes exhibiting coarse calcification, with the largest in level IV on the left side measuring 12 mm. These imaging and laboratory findings guided the clinical decision for total thyroidectomy and bilateral central neck dissection, allowing for comprehensive management of both the primary tumor and metastasis. The imaging also emphasized the need for continuous follow-up to monitor for recurrence.

### Therapeutic intervention

**Figure 1:**
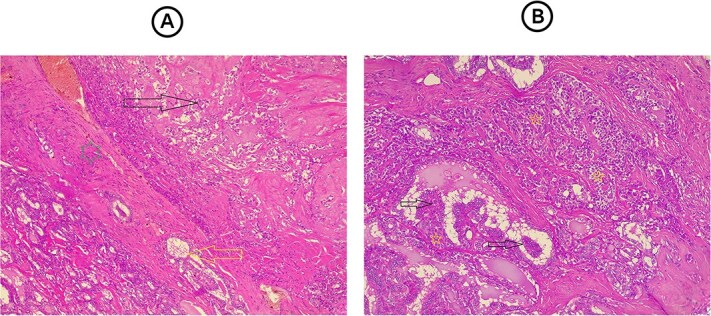
(A) Section from the thyroid tumor showing an area composed of dyscohesive plasmacytoid cells with salt-pepper chromatin and presence of eosinophilic material in between them representing amyloid material (dark arrow), while bellow it, separated by a fibrous band (green star), there is an area showing follicular epithelial cells, with nuclear features of papillary thyroid carcinoma (yellow arrow). Hematoxylin and eosin stain, 4 × 10. (B) Section from the lymph node reveal both tumor components mixed with each other, area showing papillary structures that covered by follicular epithelial cells with nuclear features of papillary thyroid carcinoma (dark arrows) with other area composed of solid nests of dyscohesive cells that representing the medullary thyroid carcinoma. Hematoxylin and eosin stain, 4 × 10.

Total thyroidectomy with bilateral central neck dissection was performed through a collar incision. Both recurrent laryngeal nerves and the parathyroid glands were achieved through meticulous dissection and careful identification of anatomical structures. Although nerve monitoring was not employed, the recurrent laryngeal nerves were preserved by maintaining their anatomical integrity and ensuring minimal manipulation. The parathyroid glands were preserved by protecting their blood supply and avoiding unnecessary handling. Despite the challenges posed by the tumor’s proximity to these structures, the careful surgical technique allowed for their successful preservation, minimizing the risk of postoperative complications. The excised tissue underwent histopathological examination (HPE), which revealed bilateral MTC with conventional PTC in the left lobe, consistent with a collision tumor, and admixture of both cancer cell types within one of the lymph nodes (Fig. 1). Gross examination revealed bilateral MTC in the right and left lobes, with a well-defined 1.5 cm nodule in the right lobe and a 2.5 cm nodule replacing the left lobe. Histologically, the MTC was multifocal and bilateral, while the PTC (0.5 cm) was unifocal in the left lobe. Both tumors showed lymph-vascular and perineural invasion, and regional lymph node involvement was present in 10 out of 39 nodes, The HPE revealed that one of the Delphian lymph nodes contained metastatic deposits from both tumors while other lymph node metastasis were all from the MTC, with extra-nodal extension and osseous metaplasia observed in the largest deposit. The collision tumor was confined to the thyroid capsule, with no evidence of extrathyroidal extension or necrosis.

### Follow-up and outcome

The patient has recovered well and will undergo regular follow-up to monitor recurrence. At the 7-week follow-up, the levels of CEA, calcitonin, and thyroglobulin were 7.02 ng/dl, 149.1 pg/ml, and 0.406 ng/ml, respectively. At the 5-month follow-up, calcitonin and thyroglobulin levels were measured at 180.2 pg/ml and 0.358 ng/ml, respectively, with no evidence of residual disease on US.

## Discussion

Literature review identified mixed thyroid carcinoma cases involving patients aged 24–64 years. Most presented with neck lumps, while some reported pain, dysphagia, or respiratory distress. Imaging (US, CT) revealed solid nodules with irregular margins, hypoechoic/hyperechoic nodules, and cystic areas, often suggesting malignancy. FNAC results varied, indicating MTC, atypical cells, or inconclusive findings. Surgical management typically involves total thyroidectomy with lymph node dissection. Histopathology confirmed mixed medullary-papillary carcinoma, sometimes with papillary or follicular components. Lymph node metastasis was observed in some cases, though many had no metastasis. Postoperative outcomes were favorable, with most patients remaining disease-free during follow-ups periods of up to 5 years. Recurrence was infrequent, and normal serum calcitonin levels indicated effective management (Table 1) [3, 4, 6–8].

**Table 1:** The characteristics of the included studies.

**First Author/years [Reference]**	**Country**	**Age**	**Sex**	**Presentation**	**Radiology** **US, CT, and MRI**	**FNA**	**Management**	**HPE** **(Thyroid lobe and LN)**		**Outcome**
Guerreiro *et al.* 2021 [3]	Portugal	60	M	NA	US: 22 mm solid nodule on left thyroid lobe with irregular margins and microcalcifications; Suspicious supraclavicular lymph node	Suggestive of MTC with supraclavicular lymph node metastasis	Total thyroidectomy with bilateral cervical lymph node dissection	Mixed medullary-papillary carcinoma	Metastases in 12/38 left and 8/10 central lymph nodes	Stable after surgery and I131 treatment; normal serum calcitonin levels; clinically well after 1 year
Wang *et al.* 2023 [4]	China	60	F	Left neck small mass	US and CT scan: hyperechoic nodule measuring ~11.9 × 9.7 mm^2^ in the left lobe of the thyroid gland	NA	Total thyroidectomy and bilateral central lymph node dissection	Mixed medullary-papillary carcinoma	No LN metastasis	After 2 years of follow-up, the patient remained healthy
Kung *et al*. 2015 [6]	Taiwan	53	F	Progressive swelling of the neck	US: isoechoic irregular 8.3 × 7.6 × 5.8 mm nodule in the right lobe of the thyroid, and a hypoechoic heterogeneous 19.2 × 18.7 × 14.0 mm nodule in the left lobe	Left lobe nodule revealed scattered atypical cells.	Radical thyroidectomy and central neck lymph node dissection	Mixed medullary-papillary carcinoma	Negative for metastatic carcinoma	NA
Raggiunti *et al*. 2014 [7]	Italy	61	F	Dysphagia and respiratory distress	US: hyperechoic nodule of 30 × 35 × 45 mm in the base of the left lobe, other hypoechoic nodules in the right lobe and isthmus	Left dominant nodule: Thy2 Bethesda	Total thyroidectomy	Mixed medullary-papillary carcinoma	NA	Clinically free of disease 5 years after surgery
Zoroquiain *et al*. 2012 [8]	Chile	24	F	Asymptomatic	US: 2-cm, irregular, solid and vascularized nodule, on the right lobe	MTC	Total thyroidectomy	Mixed medullary-papillary carcinoma	No LN dissected	After 2 years of follow-up, the patient is asymptomatic

CT, computed tomography; MRI, magnetic resonance imaging, NA, not available; PTC, papillary thyroid carcinoma; US, ultrasound; LN, lymph node; CEA, carcinoembryonic antigen; MTC, medullary thyroid carcinoma

The synchronous occurrence of MTC and PTC in the thyroid gland is extremely rare, with an annual incidence of less than 1 per 1 000 000 individuals and primarily affecting females over 45 [4]. The etiology remains unclear, with proposed hypotheses including common stem cell origin, shared tumorigenic stimuli, or coincidental occurrence. Such cases may present as mixed or collision tumors [9, 10]; here, distinct MTC and PTC lesions were found in separate thyroid regions, separated by normal tissue. This tumor is often asymptomatic and discovered incidentally. Elevated calcitonin levels can suggest the presence of a lesion [4].

When lymph node metastases occurred, they either contained both tumor components within the same lymph node or displayed one component exclusively. Distant metastases were primarily observed in the mediastinum, lung, liver, and bone. Seki *et al.* [11] demonstrated that mixed tumors have the potential to metastasize to lymph nodes, with instances of either tumor component or both present in multiple lymph nodes. Notably, in 13 out of 16 reported cases, metastasis to the same lymph node was identified.

Diagnosing mixed MTC and PTC is challenging due to overlapping histological features. Imaging studies are essential to assess thyroid involvement and lymph node metastasis. Definitive identification relies on morphological inspection, immunohistochemistry, and molecular tests on biopsy samples. Laboratory markers like calcitonin, thyroglobulin, and CEA play significant roles in confirming the diagnosis. Elevated calcitonin levels, particularly above 500 pg/ml, may indicate metastatic disease. In the current case, the patient was euthyroid with elevated levels of CEA and calcitonin [4, 12, 13].

Distinguishing benign from malignant nodules through CT and MRI cannot be reliably done but assesses tumor relationships with surrounding structures. Due to the presence of two tumor types, diagnosing and treating mixed thyroid tumors are challenging. Ryan *et al.* noted that FNA is not reliable for differentiating collision tumors. In this study, neck US revealed three distinct thyroid nodules. CT confirmed thyroid calcifications and lymph node enlargement. There are no specific radiologic features for mixed thyroid tumors [10, 13–15].

MTC metastasis is the primary cause of fatalities in combined MTC-PTC. Vigilant post-surgical monitoring includes serum tests for TG, calcitonin, and CEA every 3 months with biannual neck US to detect metastasis. The American Thyroid Association recommends radical surgery for combined MTC-PTC, typically involving total thyroidectomy and bilateral central lymph node dissection. High- or intermediate-risk PTC cases may require adjuvant radioactive iodine therapy [3, 4, 6–8, 13]. In this study, total thyroidectomy and bilateral central dissection were performed.

In conclusion, this case report highlights the exceptional rarity of mixed MTC-PTC with simultaneous lymph node involvement. Accurate diagnosis and tailored treatment require thorough preoperative assessment, including serum markers, imaging, and biopsy.
